# O073: An outbreak of norovirus strain GII.4 Sydney in a geriatric teaching hospital

**DOI:** 10.1186/2047-2994-2-S1-O73

**Published:** 2013-06-20

**Authors:** B Huttner, S Cordey, V Sauvan, L Pagani, A Iten, L Kaiser, J-L Reny, S Harbarth

**Affiliations:** 1 Infection Control Programme, Geneva University Hospitals, Geneva, Switzerland; 2 Laboratory of Virology, Geneva University Hospitals, Geneva, Switzerland; 3 Department of Internal Medicine, Geneva University Hospitals, Geneva, Switzerland

## Introduction

In 2012, several countries have reported increased Norovirus (NoV) activity attributable to the emergence of a new variant (strain GII.4 Sydney). We describe an outbreak caused by this strain in a geriatric hospital.

## Methods

Active surveillance of gastroenteritis outbreaks in a geriatric teaching hospital with 294 beds in Geneva (Switzerland) was conducted since 2008. All patients presenting ≥3 episodes of diarrhoea/day and/or ≥1 episode of vomiting not explained by other causes were reported to the infection control nurse who reviewed all cases. Viral gastroenteritis was defined as cases presenting the above mentioned symptoms plus (1) either laboratory confirmation of a viral gastroenteritis pathogen or (2) having shared a room with a case fulfilling criterion (1). A case was classified as hospital-acquired (HA) if the onset of symptoms occurred ≥48 h after admission. Symptomatic patients were put under contact and droplet precautions; environmental cleaning was intensified. Testing for NoV in stool using RT-PCR was limited to index cases. For 3 randomly selected patients symptomatic during the 2012/2013 outbreak, the NoV genome was extracted from stool samples and the ORF2 nucleotide sequence was analyzed.

## Results

During the period from 18.12.2012 to 02.01.2013 we identified 134 patients fulfilling criteria for HA gastroenteritis among 399 patients at risk during that period (attack ratio 0.34). 18/21 tested patients had a positive result for NoV, 0/15 were positive for Rotavirus, while 3 were positive for *Clostridium difficile* toxin (all 3 were also positive for NoV). 28 cases were identified among health care workers. All 3 genetically analyzed NoV samples belonged to the GII.4 Sydney strain. While outbreaks with gastroenteritis have occurred each winter season since 2008, this was the outbreak with the highest number of cases since winter 2008/2009 (total number of cases by winter: 2008/9 232 cases, 2009/10 113 cases, 2010/2011 18 cases, 2011/2012 88 cases).

## Conclusion

This study shows that the NoV GII.4 Sydney 2012 circulated in Switzerland during winter 2012/2013. An outbreak with this strain was difficult to control despite prompt instauration of infection control measures.

## Disclosure of interest

None declared.

